# Identification of two genes required for heptadecane production in a N_2_-fixing cyanobacterium *Anabaena* sp. strain PCC 7120

**DOI:** 10.1186/s13568-018-0700-6

**Published:** 2018-10-13

**Authors:** Jaimie Gibbons, Liping Gu, Huilan Zhu, William Gibbons, Ruanbao Zhou

**Affiliations:** 1 Department of Biology and Microbiology, South Dakota State University, Brookings, SD 57007 USA; 2 BioSNTR, South Dakota State University, Brookings, SD 57007 USA

**Keywords:** Cyanobacteria, Nitrogen fixation, Alkane biosynthesis, Hydrocarbons, Biofuels

## Abstract

Cyanobacteria photosynthetically produce long-chain hydrocarbons, which are considered as infrastructure-compatible biofuels. However, native cyanobacteria do not produce these hydrocarbons at sufficient rates or yields to warrant commercial deployment. This research sought to identify specific genes required for photosynthetic production of alkanes to enable future metabolic engineering for commercially viable production of alkanes. The two putative genes (*alr5283* and *alr5284*) required for long-chain hydrocarbon production in *Anabaena* sp. PCC 7120 were knocked out through a double crossover approach. The knockout mutant abolished the production of heptadecane (C_17_H_36_). The mutant is able to be complemented by a plasmid bearing the two genes along with their native promoters only. The complemented mutant restored photosynthetic production of heptadecane. This combined genetic and metabolite (alkanes) profiling approach may be broadly applicable to characterization of knockout mutants, using N_2_-fixing cyanobacteria as a cellular factory driven by solar energy to produce a wide range of commodity chemicals and drop-in-fuels from atmospheric gases (CO_2_ and N_2_ gas) and mineralized water.

**Electronic supplementary material:**

The online version of this article (10.1186/s13568-018-0700-6) contains supplementary material, which is available to authorized users.

## Introduction

Oil reserves worldwide are limited, and as prices have risen, renewable fuels have become increasingly important. Is there a biofactory that can convert carbon dioxide, water, and sunlight into fuels? Yes, several species of cyanobacteria are known to produce and secrete low levels of alkanes and alkenes using carbon dioxide, water, and sunlight (Schirmer et al. 2010).

Cyanobacteria provide numerous advantages as living “biofuel factories”. As photosynthetic organisms, they remove CO_2_ from the atmosphere to form usable carbon products (sugars, isoprenoids, fatty acids, amino acids, etc.) that support cell growth and maintenance. Fatty acids have been shown to participate in an active recycling process within the cell membrane (Kaczmarzyk and Fulda 2010). Fatty acids activated by acyl-CoA synthetase are incorporated in the membrane and are subsequently degraded and released into the pool of free intracellular fatty acids before becoming re-activated. This continual membrane-lipid recycling may serve a vital role in cyanobacterial adaptation to diverse environments and changing conditions (Kaczmarzyk and Fulda 2010). It has also been observed that cyanobacteria convert fatty acids to alkanes and alkenes via a reduction-decarbonylation pathway (Schirmer et al. 2010). Thus, cyanobacteria provide an autotrophic platform to produce petroleum-replacing chemicals that could be harnessed to also reduce greenhouse gas (GHG) emissions from CO_2_ emitting facilities, such as ethanol plants or coal-fired power plants.

Another advantage of cyanobacteria as a fuel producing platform is their minimal nutrient requirements. Many species can fix nitrogen aerobically (Bergman et al. 1997; Berman-Frank et al. 2003; Fay 1992; Zehr 2011) and have minimal requirements for trace nutrients, meaning that their primary requirements are water, sunlight, and CO_2_ (Ruffing 2011). In addition, the genomes and biochemical pathways of these autotrophic cyanobacteria are widely understood. Combined with their ease of genetic manipulation, this knowledge provides a firm foundation for genetically modifying cyanobacteria to direct carbon sources towards fuel production (Halfmann et al. 2014a, b; Lu 2010; Peralta-Yahya et al. 2012; Posewitz 2014; Ruffing 2011; Savakis and Hellingwerf 2015). Moreover, cyanobacteria have greater biomass production and photosynthetic efficiency compared to terrestrial biofuel crops, which also require use of arable land, thus impacting the food supply (Huang et al. 2010).

Heptadecane (C_17_H_36_) and pentadecane (C_15_H_32_) are the most commonly observed alkanes produced by cyanobacteria and are hypothesized to be derived from octadecanoic acid (C_18_H_36_O_2_) and hexadecanoic acid (C_16_H_32_O_2_), respectively (Coates et al. 2014; Schirmer et al. 2010). Schirmer et al. (2010) reported that the pathway consists of an acyl–acyl protein reductase (AAR) and an aldehyde decarbonylase (ADO) to form alkanes and alkenes from generally even-numbered fatty aldehyde. Thus, following the “C_n−1_” rule, odd-numbered alkanes are the typical products.

After the initial discovery of the *aar*/*ado* genes in *Synechococcus elongatus* and their involvement in hydrocarbon production (Schirmer et al. 2010), studies have used phylogenetic analysis combined with hydrocarbon profiling to identify orthologs of *aar/ado* in other cyanobacterial species (Coates et al. 2014; Liu et al. 2013). The AAR/ADO alkane biosynthesis pathway described by Schirmer is one of two hydrocarbon biosynthesis pathways operating in cyanobacteria (Mendez-Perez et al. 2011; Schirmer et al. 2010). The second pathway is the α-olefin biosynthesis (OLS) pathway (Zhu et al. 2018), which converts fatty acids into hydrocarbons via an elongation decarboxylation mechanism (Mendez-Perez et al. 2011). The AAR/ADO and OLS pathways are readily distinguishable by their products: alkane/alkenes or α-olefins, respectively (Coates et al. 2014; Mendez-Perez et al. 2011; Schirmer et al. 2010). Coates et al. (2014) reported that while all cyanobacterial species appear to be able to produce alkanes, a strain’s possession of the AAR/ADO or OLS pathway to produce alkanes is mutually exclusive in available cyanobacterial genomes, suggesting an unknown selective pressure for retaining either pathway, but not both. The AAR/ADO pathway is most prevalent among sequenced cyanobacteria (122 of 139) (Coates et al. 2014). Cyanobacterial species containing the AAR/ADO pathway predominantly produce heptadecane and branched alkanes (e.g., 7-methylheptadecane) (Coates et al. 2014; Schirmer et al. 2010).

Further investigations into hydrocarbon biosynthesis by cyanobacteria have used computational analysis such as microarray data and RNAseq to understand expression of *aar/ado* (Mitschke et al. 2011). Mitschke et al. showed that *ado* (*sll0208*) and *aar* (*sll0209*) had different expression levels under all conditions (high light, CO_2_ depletion, normal, and darkness). The *ado* expression did not vary significantly while *aar* had clear response to the conditions (with expression levels from highest to lowest being high light, CO_2_ depletion, normal, and darkness). Other research has overexpressed or introduced these genes (*aar/ado*) into other species with the aim of enhancing alkane production. Overexpression of *aar* in *S. elongatus* increased alkane production approximately twofold (Kaiser et al. 2013). Overexpression of both native copies of *aar/ado* in *Synechocystis* sp. PCC 6803 doubled alkane production compared to the parent strain, while overexpression of only one of the genes (either *aar* or *ado*) resulted in no significant changes in alkane production (Wang et al. 2013). Heterologous overexpression of *aar/ado* in *Synechocystis* sp. PCC 6803 also doubled alkane production when the genes were overexpressed simultaneously (Wang et al. 2013). Yoshino et al. (2015) demonstrated that though the AAR/ADO and OLS pathways are not natively observed to exist together in a single cyanobacterial species, the OLS-containing strain *Synechococcus* sp. NKBG15041c was able to produce heptadecane by expressing the *aar/ado* genes from *S. elongatus* PCC 7942. This research showed that heptadecane production levels in *Synechococcus* sp. NKBG15041c varied according to the expression levels of *aar/ado*, with production being the highest when the transformant carried a homologous promoter to the native *aar/ado* genes (Yoshino et al. 2015).

In this work, we identified the *aar/ado* genes in a heterocyst-forming cyanobacterium *Anabaena* sp. PCC 7120 through BLAST-P alignment with *S. elongatus* PCC 7942_orf1593 (*aar*) and orf1594 (*ado*), the *aar/ado* genes initially identified in Schirmer’s study (Schirmer et al. 2010). We aimed to directly verify that these genes are required for hydrocarbon production in vivo. Our approach was to simultaneously knock out both genes to determine the knockout mutant’s phenotype, and then re-insert the functional genes back to the knockout mutant for testing complementation. When the alkane genes were initially identified, they were believed to be part of an operon (Schirmer et al. 2010). Later research using differential RNA sequencing for genome-wide mapping of transcriptional start sites (TSS) in *Synechocystis* PCC 6803 revealed that *aar* and *ado* possess their own TSS (Mitschke et al. 2011). Subsequent research identified three promoters involved in controlling expression of *aar* and *ado* (Klahn et al. 2014). One promoter controls *aar* while two promoters (a proximal and distal promoter) control *ado* (Klahn et al. 2014).

For the complement plasmids in our study, we created constructs in which *alr5283*–*84* were placed under a combination of native and the *glnA* (*alr2328*) promoters. Through varying the promoter systems in the complement study, we hoped to gain further insight into the control mechanisms behind the alkane genes’ transcriptional regulation. Our results demonstrated that the native promoter system was the only one able to complement the knockout mutant, highlighting the importance and underlying complexity of the native three-promoter system.

## Materials and methods

### Bacterial strains and plasmids

*Escherichia coli* strains Top10 (Invitrogen) and NEB10β (New England Biolabs) were used for plasmid construction and maintenance. *E. coli* strains were grown in Luria–Bertani broth. Antibiotic concentrations used for *E. coli* strains were 100 µg mL^−1^ ampicillin (Ap), 50 µg mL^−1^ kanamycin (Km), 25 µg mL^−1^ chloramphenicol (Cm), 100 µg mL^−1^ spectinomycin (Sp), and 10 µg mL^−1^ erythromycin (Em). Antibiotic concentrations used for cyanobacterial mutant strains were 100 µg mL^−1^ neomycin (Nm), 10 µg mL^−1^ Sp, 10 µg mL^−1^ Em or except where noted.

### Construction of aar/ado knockout plasmid pZR935

To disrupt the *alr5283*–*5284* sequence in *Anabaena* 7120 genome, plasmid pZR935 was created (details see Table 1) and transferred to *Anabaena* 7120 via conjugative transformation to replace chromosomal *alr5283*–*84* with the disrupted *alr5283*–*84* sequence via double recombination. Briefly, *alr5283*–*5284* and flanking sequences were PCR amplified with specific primers ZR241, ZR242 (Table 2) from *Anabaena* 7120 genomic DNA, cloned into pCR^®^2.1-TOPO^®^ vector (TOPO TA Cloning^®^ kit, Invitrogen), creating pZR932. Next, site directed mutagenesis using primers ZR243, 244 introduced a *Not*I site into *alr5283* within pZR932, creating pZR933. Then, the mutated *alr5283*–*84* sequence was excised from pZR933 and transferred to the vector pZR824, creating pZR934. Finally, the 3′ end of *alr5283* and the 5′ end of *alr5284* were excised from pZR934 using restriction enzymes *Not*I and *Nhe*I; a promoter-less GFP-Spec cassette from pZR666 was inserted into *Not*I and *Xba*I digested pZR934, creating pZR935 (details see Additional file 1: Fig. S1).

**Table 1:** Plasmids and bacterial strains used in this study

Plasmid or strain	Relevant characteristic(s)	Source or references
Plasmids		
pAM1956	Promoter-less GFPmut2 cloning vector	Yoon and Golden (1998)
pRL271	Cm^r^/Em^r^. Integration vector	Cai and Wolk (1990)
pRL278	Km^r^/Nm^r^, integration vector	Cai and Wolk (1990)
pRL443	Ap^r^; conjugal plasmid	Elhai et al. (1997)
pRL623	Cm^r^; helper plasmid	Elhai et al. (1997)
pZR606	Km^r/^Sp^r^, integration vector	Chen et al. (2015)
pZR618	Ap^r^; T7-MCS-F_2_-H_6_	Chen et al. (2012)
pZR666	Km^r^/Sp^r^, MCS-*gfp*-MCS cassette, annealed oligonucleotides LK2406/LK2407 ligated to *Afl*II digested pZR606 to produce pZR666	This study
pZR670	Cm^r^/Em^r^; expression vector for *Anabaena*	Chen et al. (2015), Xu et al. (2015)
pZR824	Km^r^; integration vector, annealed oligonucleotides ZR165/ZR166 ligated to *Bgl*II-*Spe*I digested pRL278 to produce pZR824	This study
pZR932	Km^r^/Ap^r^; *alr5283*–*84* ORF amplified by PCR with primers ZR241 and 242 from *Anabaena* 7120 chromosomal DNA and cloned into pCR2.1-TOPO vector	This study
pZR933	Km^r^/Ap^r^; site-directed mutagenesis using primers ZR243 and 244 to introduce *Not*I site into *alr5283*–*84* within pZR932	This study
pZR934	Km^r^; *Bam*HI and *Avr*II digested *alr5283*–*84* ORF from pZR933 ligated to *Bgl*II and *Spe*I digested pZR824	This study
pZR935	Km^r^/Sp^r^; *Not*I and *Xba*I promotor-less GFP-Sp^r^ cassette from pZR666 ligated to *Not*I and *Nhe*I digested pZR934 to disrupt *alr5283*–*84*	This study
pZR2222	Km^r^/Ap^r^; Primers ZR1584 and ZR1585 PCR amplified 1921 bp Cm^r^/Em^r^ cassette (*Nsi*I/*Nde*I-*Nhe*I/*Bam*HI-Em^r^/Cm^r^ cassette-*Eco*RV/*Bgl*II/*Xho*I/*Xma*I/*Sma*I) from pRL271 ligated to pCR2.1-TOPO to produce pZR2222	This study
pZR2223	Cm^r^/Em^r^; expression vector, *Bam*HI/*Xma*I cut out 1.9 kb Cm^r^/Em^r^ cassette from pZR2222 and ligated to *Bgl*II/*Sgr*AI-digested pAM1956	This study
pZR2238	Km^r^/Ap^r^; *Aat*II/*Sal*I-P-*alr5283*-P-*alr5284*-*Kpn*I/*Xma*I PCR amplified by ZR1602 and 1603 from *A.* 7120 ligated to pCR2.1-TOPO	This study
pZR2239	Cm^r^/Em^r^; *Sal*I-P-*alr5283*-P-*alr5284*-*Xma*I from pZR2238 ligated to *Sal*I/*Xma*I cut pZR2223	This study
pZR2242	Km^r^/Ap^r^; *Nsi*I/*Nde*I-*alr5283* orf-P-*alr5284* orf-*Bam*HI PCR amplified from *A.* 7120 ligated to pCR2.1-TOPO	This study
pZR2243	Cm^r^/Em^r^; *Nsi*I-*alr5283* orf-P-*alr5284* orf-*Bam*HI from pZR2242 ligated to *Nsi*I-*Bam*HI cut pZR670	This study
pZR2244	Km^r^/Ap^r^; *Nsi*I/*Nde*I-*alr5283* orf PCR amplified by ZR1606 and 1608 from *A.* 7120; RBS-*alr5284* orf-*Bam*HI PCR amplified by ZR1609 and 1607 from *A.* 7120; PCR overlap of *Nsi*I/*Nde*I-*alr5283* orf and RBS-*alr5284* orf-*BamH*I with primers ZR1606 and 1607; PCR overlap product ligated to pCR2.1-TOPO	This study
pZR2248	Cm^r^/Em^r^; *Nsi*I-*alr5283* orf-RBS-*alr5284* orf-*Bam*HI from pZR2244 ligated to *Nsi*I-*Bam*HI cut pZR670	This study
Bacterial strains		
TOP10	*E. coli* cloning host	Invitrogen
NEB10β	*E. coli* cloning host	New England Biolabs
WT7120	*Anabaena* sp. PCC 7120 wild-type strain	This study
DR935	Sp^r^; *alr5283*–*84* double knockout mutant	This study
DR935(pZR2239)	Sp^r^, Em^r^; DR935 containing pZR2239 for complementation study	This study
DR935(pZR2248)	Sp^r^, Em^r^; DR935 containing pZR2248 for complementation study	This study
DR935(pZR2243)	Sp^r^, Em^r^; DR935 containing pZR2243 for complementation study	This study

Ap^r^: ampicillin resistance; Sp^r^: spectinomycin resistance; Nm^r^/Km^r^: neomycin–kanamycin resistance; Cm^r^/Em^r^: chloramphenicol–erythromycin resistance; F_2_: two FLAG epitopes; MCS: multiple cloning sites; RBS: ribosome-binding site; P-: promoter; orf: open reading frame

**Table 2:** Primers used in this study

Primers	Oligonucleotide sequences (5′ → 3′)	Description
ZR165	GATCTCCCGGGCTAGCGGCCGCAATTGACGTCTCGAGA	Annealed oligonucleotides ZR165/ZR166 ligated to *Bgl*II-*Spe*I digested pRL278 to produce pZR824
ZR166	ctagTCTCGAGACGTCAATTGCGGCCGCTAGCCCGGGA	
ZR241	tggaTCCAACTCTACAGGAATTGTCTG	ZR241,242 primer pair amplifying *alr5283*–*84* ORF (2.7 kb); in knockout mutant with GFP-Sp^r^ cassette, primers amplify 4.7 kb
ZR242	tcctAGGAATTGGTATTGGGGATTG	
ZR243	CAAAAAGCGGCcGCTGAAGGTAAAATC	ZR243,244 primer pair for site-directed mutagenesis to introduce *Not*I site at 997 bp of *alr5283*–*84* region
ZR244	TTTACCTTCAGCgGCCGCTTTTTGG	
ZR1584	atgcatatgctagcgacgtcggATCCCTTAACTTACTTATTAAATAATTTATAG	Primers ZR1584/ZR1585 PCR amplified 1921 bp Cm^r^/Em^r^ cassette (*Nsi*I/*Nde*I-*Nhe*I/*Bam*HI-Em^r^/Cm^r^ cassette-*Eco*RV/*Bgl*II/*Xho*I/*Xma*I/*Sma*I) from pRL271 ligated to pCR2.1-TOPO to produce pZR2222
ZR1585	tccCGGGAAGTATCCAGCTCGAGATC	
ZR261	CAAGAATTGGGACAACTCCAGTG	ZR261, 1602 primer pair for verifying insertion of *P*-*alr5283*-*P*-*alr5284* in pZR2223 shuttle vector (construction pZR2239)
ZR1602	tgacgtcGACTCCAAAAATCAGCAGATTTCC	
ZR1603	tcccgggtacCTAAACCAGCAGTGGTCTAAACC	ZR1602, 1603 primer pair amplifying P-*alr5283*-*P*-*84* (2.2 kb); amplification retains promoters for both genes
ZR1606 ZR1607 ZR1608 ZR1609	atgcatATGCAGCAGGTTGCAGCCGATTTAG tggatCCTAAACCAGCAGTGGTCTAAACC CATGGtatatctccttcttTTAAGCTGCTGTAAGTCCGTAGGCT GACTTACAGCAGCTTAAaagaaggagatataCCATGTTTGGTCTAATTGG	ZR1606,1607 primer pair amplifying *alr5283*–*84* (2 kb); amplification includes promoter for *alr5284* only ZR1606-1608-1609-1607 overlap PCR (1.7 kb) (1) Primer pair ZR1606, 1608 amplify *alr5283* ORF (2) Primer pair ZR1609, 1607 amplify *alr5284* ORF (3) Primer pair ZR1606, 1607 using template fragments from steps 1 and 2 for PCR overlap, combining the fragments (1.7 kb)
LK2406	TTAAGGGCCCGGGAGATCTAGACCGGTACTAGTC	Annealed oligonucleotides LK2406/LK2407 ligated to *Afl*II digested pZR606 to produce pZR666; *Apa*I-*Xma*I-*Bgl*II-*Xba*I-*Age*I-*Spe*I multiple cloning sites (MCS)
LK2407	TTAAGACTAGTACCGGTCTAGATCTCCCGGGCCC	

Sp: spectinomycin resistance; ORF: open reading frame; GFP: green fluorescent protein; MCS: multiple cloning sites, RBS: ribosome-binding site, P-: promoter, PCR: polymerase chain reaction

### Knocking out alr5283–5284 in *Anabaena* sp. PCC 7120

Tri-parental mating was initiated by mixing HB101 [pRL623 + 443] with *E. coli* 10β containing the cargo plasmid pZR935. The *E. coli* strains were combined in a single 1.5 mL tube and set at room temperature for 30 min to allow the strains to mate. Following mating, *Anabaena* 7120 was added to the mating mixture. Cultures of *Anabaena* 7120 were grown in BG11 medium and incubated at 30 °C, 120 rpm, under continuous light (60 µmol E m^−2^ s^−1^) for 7 days until the culture reached an OD_700_ of 0.5. Ten millilitre of the culture was harvested (4000×*g*, 10 min), and the pellet was washed with 1 mL BG11. Following a second centrifugation (12,000×*g*, 1 min), the pellet was resuspended in 100 µL BG11. This was added to the *E. coli* mixture containing the cargo plasmid for transformation, the helper plasmid pRL623, and the conjugal plasmid pRL443 (Elhai et al. 1997). The cyanobacteria and *E. coli* were allowed to mate for 1 h. Then, the solution was plated on a nitrocellulose membrane on BG11 agar supplemented with 5% LB and incubated at 30 °C under light for 2 days. Next, the membrane was transferred to a BG11 plate containing the antibiotic to select for transformed *Anabaena* 7120. Plates were incubated at 30 °C under light until single colonies formed. On a weekly basis, membranes were transferred to new BG11 antibiotic plates. More details were described in Target Gene Inactivation in Cyanobacterium *Anabaena* sp. PCC 7120 (Chen et al. 2016).

Conjugal transformation of pZR935 to *Anabaena* 7120 to achieve a double crossover was first verified by distinguishing single crossover colonies via colony PCR (primers listed in Table 2). Single crossover recombination colonies named SR935 were then grown in 10 mL BG11 plus spectinomycin for 1 week. Then, 1 mL of SR935 was sonicated for 10 min until all filaments were separated into single cells (visualized under a microscope). The sonicated culture was harvested (13,000×*g*, 1 min), and the pellet was resuspended in 100 µL BG11. The suspension was plated on BG11 plus spectinomycin and 5% sucrose. Spectinomycin selected for transformed *Anabaena* 7120 over wildtype while sucrose selected against single crossovers due to pZR935 serving as a *sacB*-based suicide vector. After 1 week’s growth under light at 30 °C, single colonies appeared and were verified to be double recombinants via colony PCR; double crossover recombinants are henceforth referred to as the *alr5283*–*alr5284* knockout mutant named DR935.

### Complementation of Alr5283–Alr5284 knockout mutant

To complement the knockout mutant (DR935), three plasmids (pZR2239, pZR2248, and pZR2243) were constructed containing *alr5283*–*84* under different promoter systems. Briefly, pZR2239 contained the genes both under their native promoters. In plasmid pZR2248, both genes were under control of the *glnA (alr2328*) promoter, though each gene contained its own ribosomal binding site. Plasmid pZR2243 contained *alr5283* under control of the constitutive glutamine synthetase (*glnA*) promoter while *alr5284* remained under control of its native promoter. Table 1 provides further details of the plasmids and bacterial strains used in this study. Q5-High-Fidelity DNA Polymerase (NEB) was used for all PCR amplifications. Cloned PCR products and mutated genes were verified by Sanger DNA sequencing at GenScript. All cloning enzymes such as restriction endonucleases were purchased from NEB. All PCR primers synthesized at Integrated DNA Technologies (IDT) are listed in Table 2.

Conjugal transformation of individual complement plasmids pZR2239, pZR2248, or pZR2243 to DR935 was performed using the same conjugation method detailed above. After obtaining individual colonies on the antibiotic selection plate, successful complements were verified by performing colony PCR with individual colonies. Verified complements carrying plasmids pZR2239, pZR2248, and pZR2243 were named as DR935(pZR2239), DR935(pZR2248), and DR935(pZR2243), respectively.

### Headspace sample collections and extractions

Wildtype *Anabaena* 7120; DR935; and the complementing strains, DR935(pZR2239), DR935(pZR2248), and DR935(pZR2243), were grown in 100 mL BG11 for 17 days with an initial OD_700_ of 0.03. Cultures were incubated at 30 °C, 120 rpm at an aeration rate of 100 mL min^−1^. A resin column inserted into the rubber stopper sealing each flask was used to capture hydrocarbons in the headspace (Halfmann et al. 2014a); each resin column contained 0.12 g Supelpak-2SV resin (Sigma-Aldrich). During the incubation period, hydrocarbons were extracted from the resin columns on day 5.

To extract hydrocarbons bound to the resin Supelpak-2SV, the resin was transferred to 1.5 mL eppendorf tubes, and 1 mL pentane containing 5 µg mL^−1^ tetracosane as an internal standard was added to the resin. The resin and pentane mixture was vortexed for 1 min and left to sit for 10 min. After 10 min, each sample was vortexed for 10 s and pentane was removed and stored in a 2 mL glass vial for GC/MS analysis. Ten µg mL^−1^ standards of pentadecane, 1-pentadecene, heptadecane, and 1-heptadecene (TCI) were analyzed by GC/MS for comparison purposes.

### Gas chromatography/mass spectrometry (GC/MS) analysis of hydrocarbon samples

Hydrocarbon extractions from the cultures were analyzed using GC/MS (Agilent 7890A/5975C) at the Functional Genomics Core Facility of South Dakota State University. One-microliter injected samples were separated by an HP-5MS column (35 m × 250 µm × 0.25 µm), with H_2_ serving as the carrier gas. The oven temperature was initially held at 145 °C for 2 min, increased 5 °C min^−1^ until 180 °C was reached, and then increased by 40 °C min^−1^ to 300 °C. Total run time was 14 min. Compounds were identified using the NIST MS library v2.0 and further verified by authentic standards.

The initial GC/MS analysis of the headspace samples from *Anabaena* 7120 used 50–500 m/z full scan, which identified heptadecane production. Based on the spectra obtained in the full scan of heptadecane and the internal standard tetracosane, we created an selected ion monitoring (SIM) method to use for future analysis of wildtype, DR935, and complement strains DR935(pZR2239), DR935(pZR2248), and DR935(pZR2243). SIM was chosen because it is more selective and it provides a better signal for heptadecane. The GC/MS SIM method used selected ion monitoring parameters of (57, 71, and 240) and (57, 71, and 338) for heptadecane and tetracosane, respectively. Heptadecane had a retention time of 6.31 min; tetracosane had a retention time of 11.8 min.

### Chlorophyll content analysis

Chlorophyll content was quantified at OD_665_ following the previously detailed method (Houmard and de Marsac 1988).

### GenBank access to genes

The two genes knocked out for the alkane study, *alr5283* and *alr5284*, can be found through GenBank using Accession Numbers NC_003272.1:6303216-6303911 and NC_003272.1:6304154-6305173, respectively.

## Results

### Identification of heptadecane emitted from *Anabaena* 7120

**Fig. 1:**
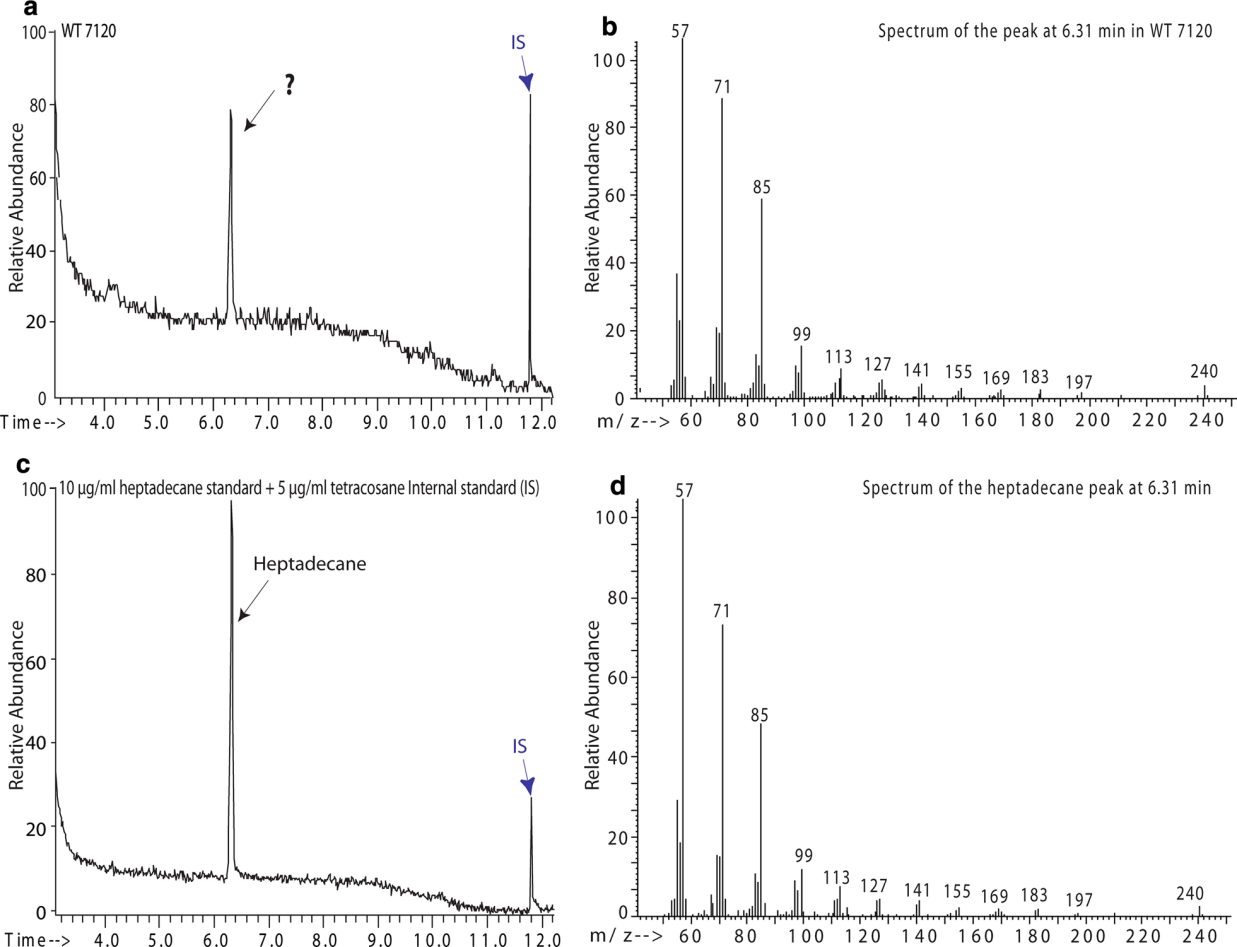
Identification of heptadecane produced by *Anabaena* 7120. **a** GC/MS chromatograph of the volatile metabolites from *Anabaena* 7120 cultures. A peak at the retention time of 6.31 min (black arrow) found in *Anabaena* matches the heptadecane standard (**c**). **b**, **d** Mass spectra of the 6.31 min peaks display the fragmentation pattern for the compound in *Anabaena* and the heptadecane standard, respectively. Five μg mL^−1^ tetracosane serves as an internal standard (IS, blue arrow)

A GC/MS analysis of volatile compounds emitted from wildtype *Anabaena* 7120 revealed a prominent peak at retention time 6.31 min (Fig. 1a). The peak was identified as heptadecane (C_17_H_36_) by the mass spectral library. To confirm the identity of the hydrocarbon peak, authentic standards for pentadecane, 1-pentadecene, heptadecane, and 1-heptadecene were analyzed by GC/MS. Heptadecane had the same retention time (6.31 min) as the wildtype compound (Fig. 1c) and showed the same fragmentation pattern as the wildtype compound (comparing Fig. 1d to b). Thus, we concluded that the major hydrocarbon peak detected in *Anabaena* 7120 is heptadecane (C_17_H_36_).

### Bioinformatics analysis to identify hydrocarbon biosynthesis genes

**Fig. 2:**
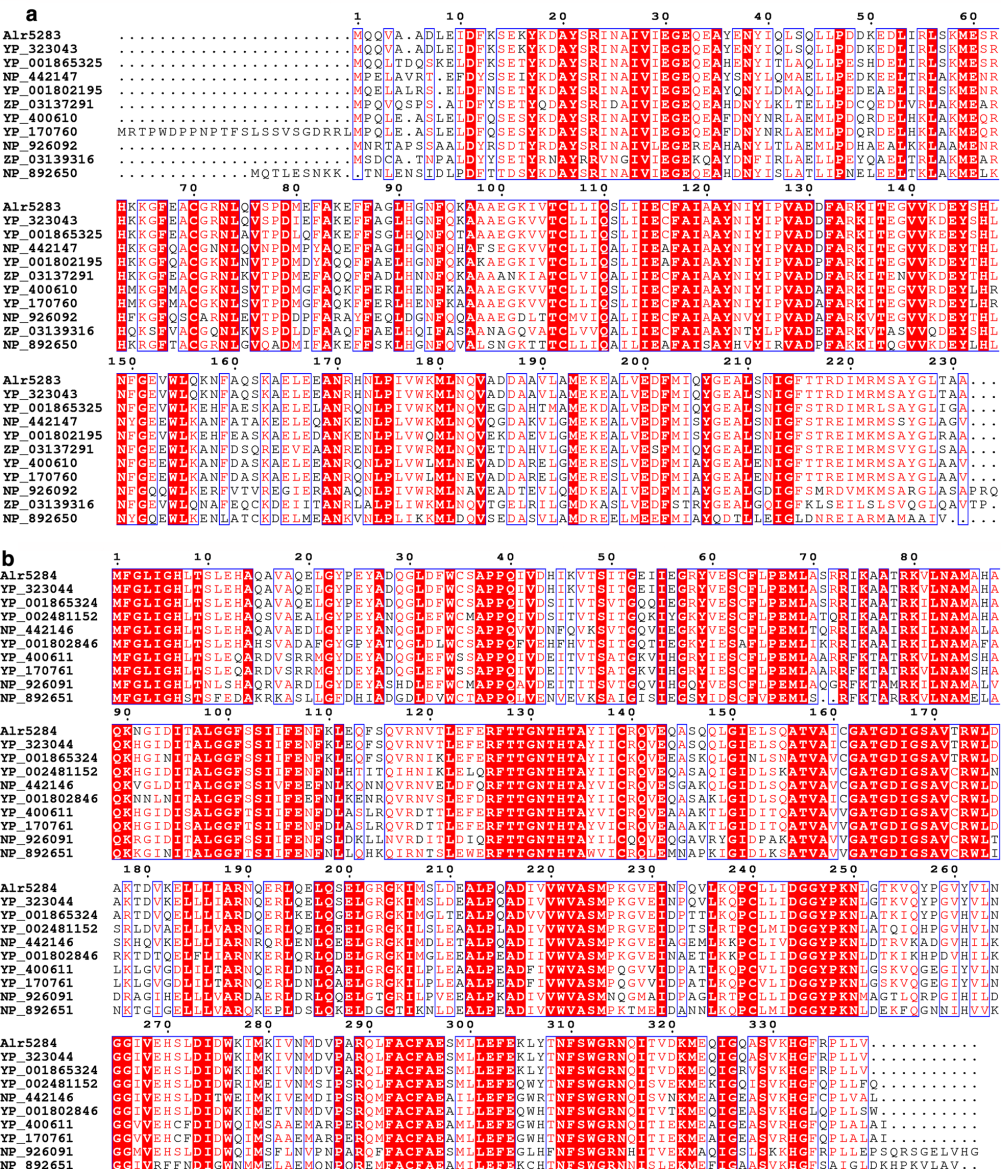
Identification of the aldehyde decarbonylase and acyl-ACP reductase *Anabaena* 7120. **a** Multiple sequence alignment of aldehyde decarbonylase in cyanobacteria. **b** Multiple sequence alignment of acyl-ACP reductase in cyanobacteria. *Anabaena* 7120 consists of aldehyde decarbonylase (Alr5283, Accession No. BAB76982) and acyl-ACP reductase (Alr5284, Accession No. BAB76983). Homologs used in the multiple sequence alignment include YP_323043 and YP_323044 in *Trichormus variabilis* ATCC 29413; YP_001865325 and YP_001865324 in *Nostoc punctiforme* PCC 73102; NP_442147 and NP_442146 in *Synechocystis* sp. PCC 6803; YP_001802195 and YP_001802846 in *Cyanothece* sp. ATCC 51142; ZP_03137291, ZP_03139316 and YP_002481152 in *Cyanothece* sp. PCC 7425; YP_400610 (Synpcc7942_1593) and YP_400611 (Synpcc7942_1594) in *Synechococcus elongatus* PCC 7942; YP_170760 and YP_1707601 in *Synechococcus elongatus* PCC 6301; NP_926092 and NP_926091 in *Gloeobacter violaceus* PCC 7421; and NP_892650 and NP_892651 in *Prochlorococcus marinus* subsp*. pastoris* str. CCMP1986, respectively. Sequence alignment was made using an online program MultAlin (Corpet 1988). The figure was generated by an online program ESPript 3.0 (Robert and Gouet 2014)

Hydrocarbon biosynthesis genes have been identified in the cyanobacterial species *S. elongatus* PCC 7942 (Schirmer et al. 2010). Hydrocarbon biosynthesis genes have not yet been verified in any N_2_-fixing cyanobacteria such as *Anabaena* 7120. To identify the genes required for heptadecane production in *Anabaena* 7120, a BLAST-P search using the representative proteins from *S. elongatus* against the publically available *Anabaena* 7120 genome database was performed to identify putative alkane biosynthesis proteins and their respective genes. The protein sequence alignment revealed Alr5283 and Alr5284 from *Anabaena* 7120 were homologous to ADO and AAR from nine cyanobacterial species known to produce heptadecane and pentadecane (Schirmer et al. 2010) (Fig. 2a, b, respectively).

### Construction of DR935

**Fig. 3:**
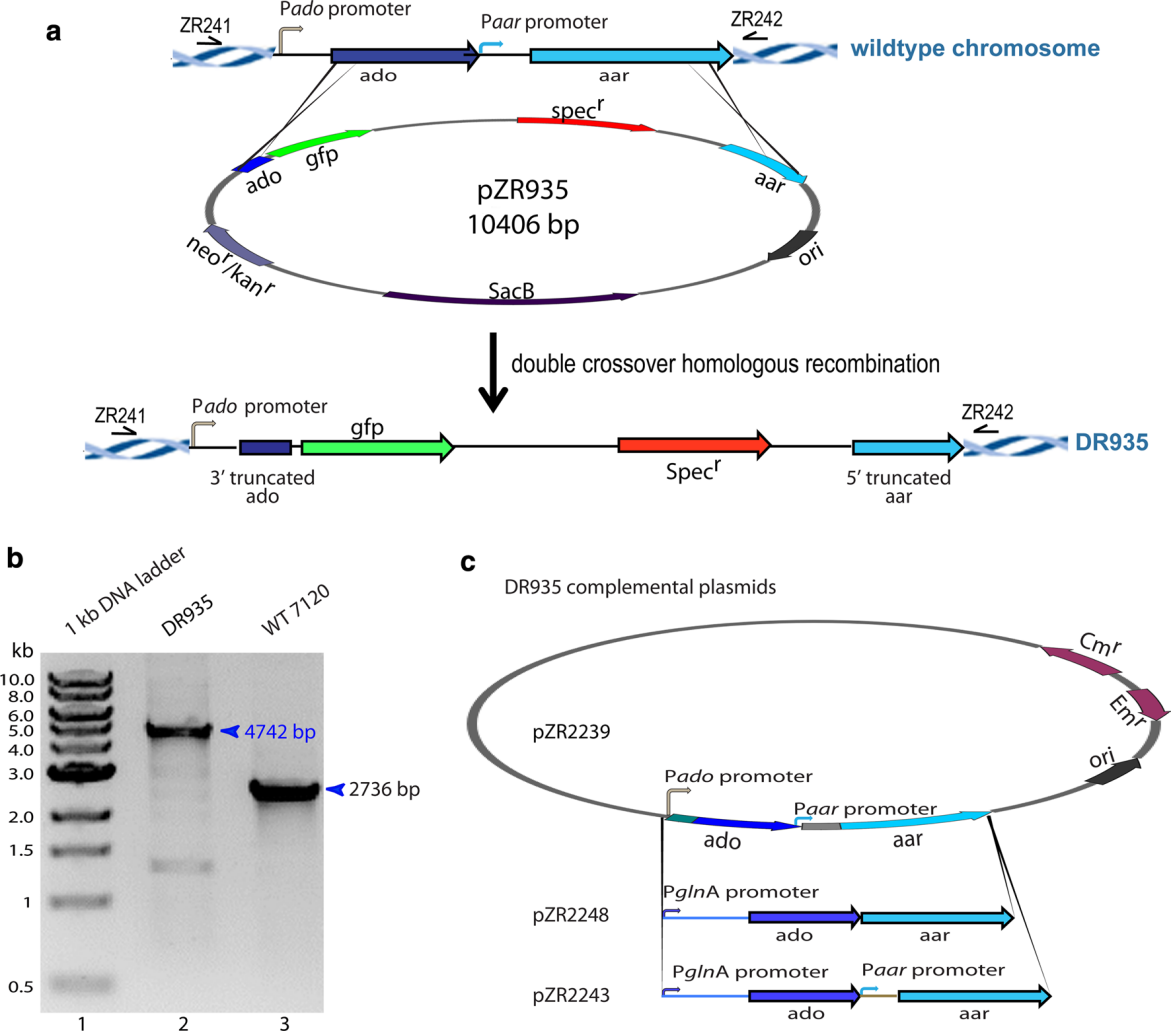
Construction of Alr5283-Alr5284 knockout mutant (DR935) and its complemented strains in *Anabaena* 7120. **a** 3′ deletion of *ado* (*alr5283*) and 5′ deletion of *aar* (*alr5284*) created by inserting a *gfp*-*spec* cassette between *alr5283*–*84* in *Anabaena* 7120 chromosome via double recombination with knockout plasmid pZR935. **b** PCR verification of DR935. Wildtype *Anabaena* 7120 had the intact *alr5283*–*84* gene sequence, which has a length of 2.7 kb when amplified by ZR241, 242 (lane 3). DR935 contained the *gfp*-*spec* cassette inserted in the *alr5283*–*84* gene sequence, making the amplified gene sequence 4.7 kb (lane 2). **c** Complementing plasmid constructions: pZR2239 contains *ado* and aa*r* both under control of their native promoters, pZR2248 contains the engineered *ado*-*aar* operon under control of the constitutive *glnA* promoter, a standard ribosome-binding sequence (AAGGAGA) was introduced between *ado* and *aar* in pZR2248, and pZR2243 contains *ado* under control of *PglnA* and *aar* under control of its native promoter

The two putative genes (*alr5283*–*alr5284*) whose products may be responsible for long-chain hydrocarbon production were cloned from *Anabaena* 7120. The genes were rendered nonfunctional through deletions of the 3′ end of *alr5283* and 5′ end of *alr5284*; the native sequence of these genes was further disrupted by a *gfp*-*spectinomycin* cassette inserted between the *alr5283* and *alr5284* gene sequences (Fig. 3a and Additional file 1: Fig. S1).

Conjugative transformation of pZR935 to *Anabaena* 7120 resulted in a double crossover, which replaced the functional *alr5283*–*84* gene sequence with the disrupted gene sequence (Fig. 3a). DR935 was verified by colony PCR from the conjugation (Fig. 3b). An expected 2.7 kb PCR product using primers ZR241 and ZR242 flanking the *alr5283*–*84* gene sequence was detected in the wildtype *Anabaena* 7120 (Fig. 3b lane 3). In DR935, containing the *gfp*-*spec* cassette within the *alr5283*–*84* gene sequence, the PCR product amplified by primers ZR241 and ZR242 increases to 4.7 kb (Fig. 3b lane 2), while the 2.7 kb PCR product in wild-type *Anabaena* 7120 was under detectable. Thus, the DR935 was completely segregated, the pure double recombinant DR935 containing only the 4.7 kb amplification were obtained. Verified DR935 double mutant was used for further analysis. Three plasmids were constructed (Fig. 3c) to test complementation of DR935.

### Alr5283 and Alr5284 are responsible for heptadecane production in *Anabaena* 7120

**Fig. 4:**
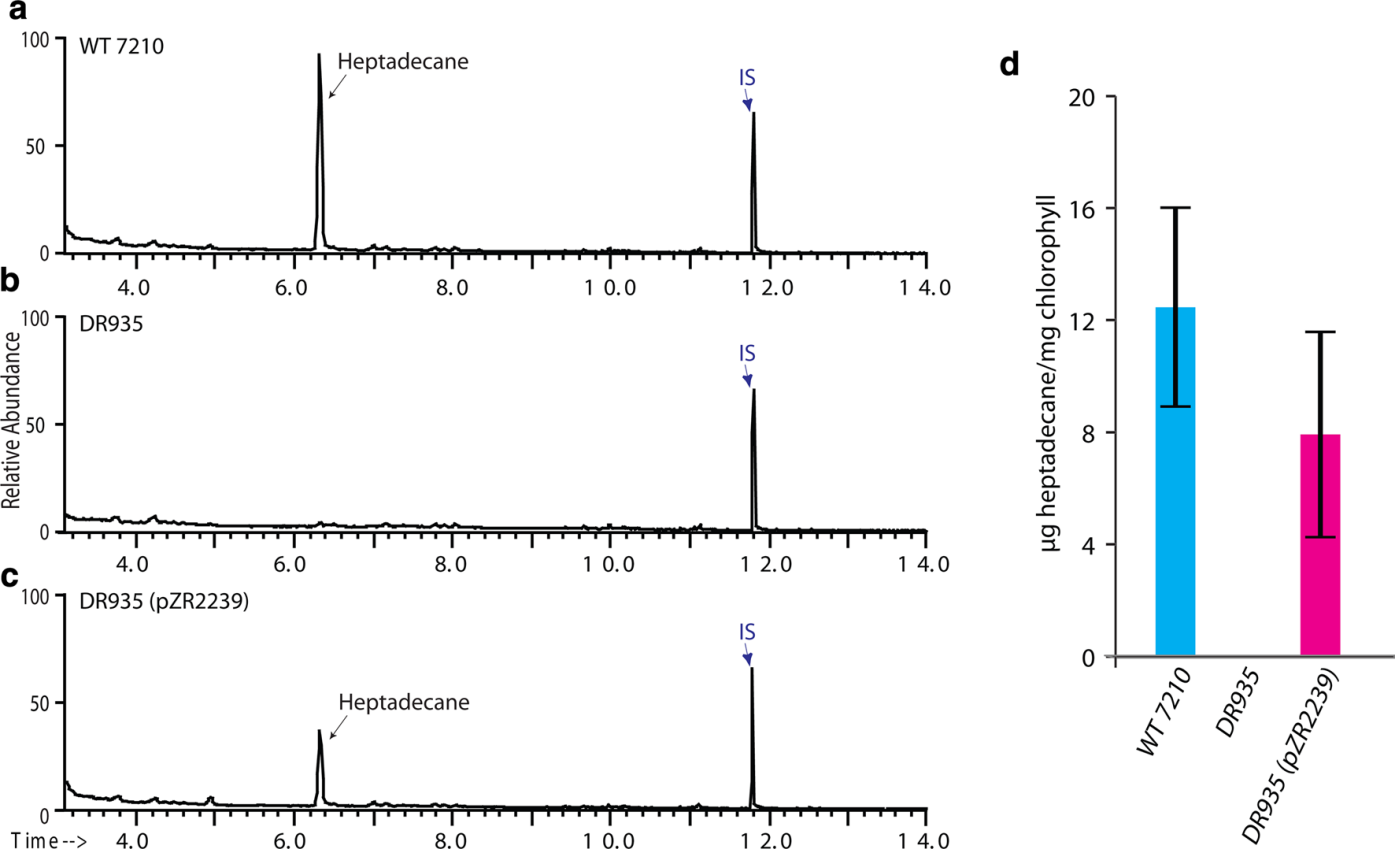
Alr5283 and Alr5284 are responsible for the heptadecane production in *Anabaena* 7120. GC/MS SIM chromatographs of the volatile metabolites from wildtype (**a**), DR935 (**b**), and DR935(pZR2239) (**c**) strains, respectively. Heptadecane naturally produced in wildtype *Anabaena* 7120 was not detected in DR935, but reemerged after the complement plasmid pZR2239 was transformed into DR935. Five μg mL^−1^ tetracosane serves as an internal standard (IS, blue arrow). **d** Total heptadecane production mg^−1^ chlorophyll of wildtype *Anabaena* 7120, DR935, and DR935(pZR2239) cultures from days 0 to 5. Heptadecane yield was calculated by measuring the total heptadecane produced from days 0 to 5 and dividing by total chlorophyll content of the 100 mL cultures measured on day 5. The culture density increased over days 0–5 but only day 5 chlorophyll content was used to make the calculation (the chlorophyll content of seed culture at day 0 is negligible). Therefore, the total heptadecane production mg^−1^ chlorophyll calculation is an underestimate

Volatile compounds emitted from wildtype, DR935, and complement strains were analyzed via GC/MS SIM. In DR935, the heptadecane peak at 6.31 min disappeared, indicating that *alr5283*–*84* are required for heptadecane production in *Anabaena* 7120 (Fig. 4b). Among the three complement plasmids constructed, only pZR2239, containing *alr5283*–*84* under control of the native 3-promoter system, was able to rescue heptadecane production in the knockout mutant DR935 (Fig. 4c). Neither pZR2248 nor pZR2243 was able to recover heptadecane production in the knockout mutant DR935 (data not shown).

### Quantification of heptadecane production in wild-type and complemented strain

We analyzed the amount of heptadecane produced from wildtype *Anabaena* 7120, DR935, and DR935(pZR2239) strains during the first 5 days of the experiment. Overall, heptadecane production was higher in the wildtype (WT 7120) compared to the complemented strain DR935(pZR2239) and undetectable in the DR935 (Fig. 4d).

**Fig. 5:**
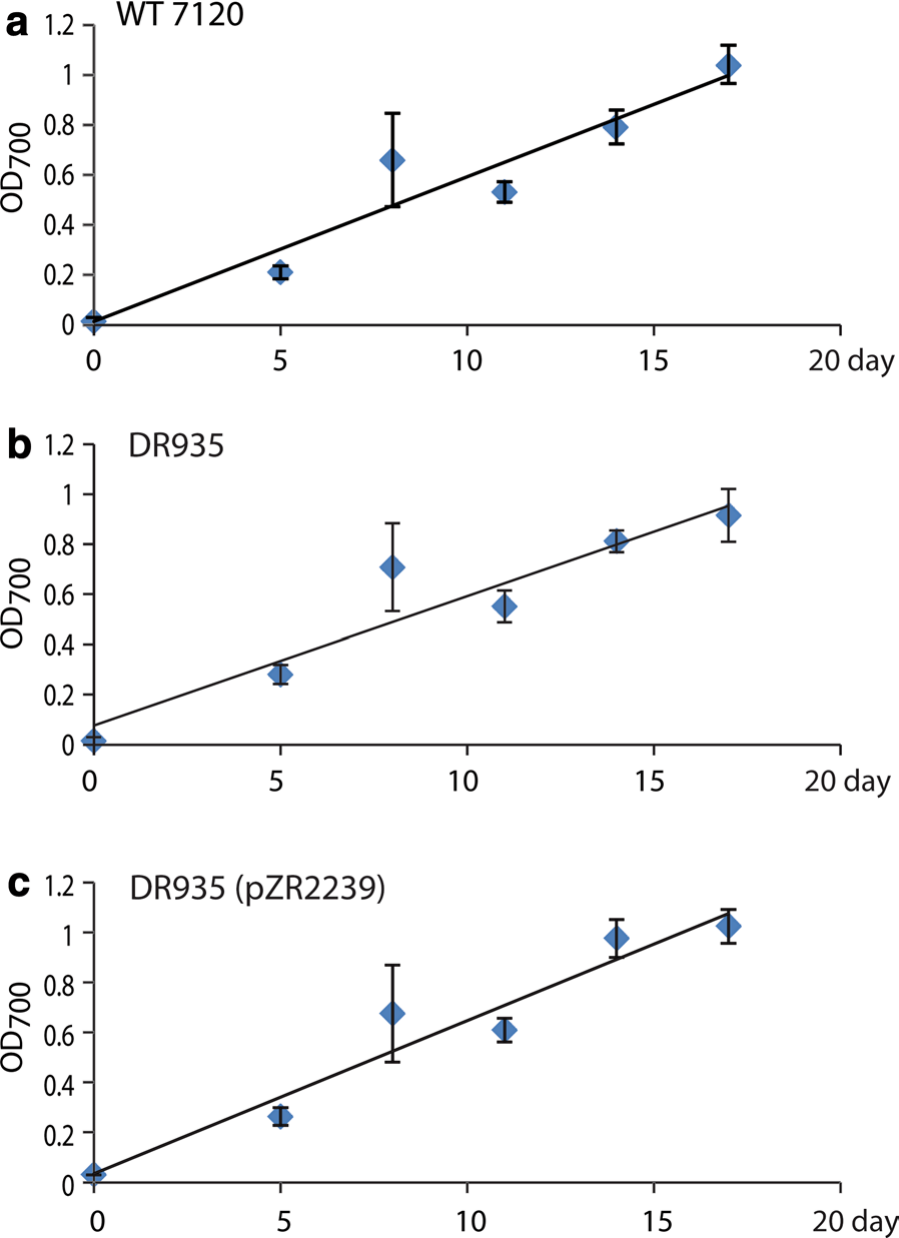
Growth phenotypic analysis from wildtype, DR935, and DR935(pZR2239) cultures grown in BG11 medium. Growth curve of wildtype *Anabaena* 7120 (**a**), DR935 (**b**), and **c** DR935(pZR2239)

Though heptadecane production differed amongst the strains, it did not appear to impact normal growth of the culture as indicated by OD measurements (Fig. 5) and visual qualitative analysis of culture growth (data not shown). These results indicate that under the growth conditions used in this study, heptadecane production does not have a significant impact on cell growth or survival.

## Discussion

In a previous study of alkane production by cyanobacteria, heptadecane and pentadecane were cited as the most commonly produced alkanes (Schirmer et al. 2010). In our work, heptadecane was the predominant volatile alkane captured by Supelpak 2SV resin (Sigma-aldrich) and detected in headspace of wildtype *Anabaena* 7120 culture. Heptadecane production was lost when *alr5283*–*84* were disrupted and re-emerged after complemented by intact a*lr5283* and *alr5284* driven by their native promoters. Thus, the enzymes Alr5283 and Alr5284 from *Anabaena* 7120 are required for heptadecane production. Our findings are partially supported by previous reports, which detected a range of hydrocarbons produced by the *ado/aar* gene products from various cyanobacteria when the genes were expressed in *E. coli* (Schirmer et al. 2010) or in *Synechocystis* sp. PCC 6803 (Wang et al. 2013). Taken together, we conclude that the ADO/AAR enzymes encoded by *alr5283*–*84* are responsible for heptadecane production from a C_18_ fatty acid substrate in *Anabaena* 7120.

### Differential expression of ADO and AAR is critical for heptadecane production

A complementation experiment was required to confirm that two genes (*ado* and *aar*), rather than a downstream gene *alr5285* (acetyl-CoA carboxylase alpha subunit), are responsible for DR935 mutant phenotype (loss of heptadecane production). The replicating plasmid pRL2833a (Wolk et al. 2007) and its derivative pZR670 (Chen et al. 2016) had been successfully used for complementing many knock-out mutants in *Anabaena* 7120 (Chen et al. 2016; Fan et al. 2005; Xu et al. 2015). The *glnA* promoter is a constitutive expression promoter and is up-regulated by nitrogen starvation (Flaherty et al. 2011). The *glnA* promoter was successfully used for complementing *alr4853* mutant in *Anabaena* 7120 (Xu et al. 2015). DR935(pZR2248) contained both genes in a two gene operon (*ado*–*aar*) under the *glnA* promoter, yet did not recover heptadecane production. Importantly, among the complement plasmids used to recover heptadecane production in the *ado/aar* knockout mutant DR935, only the plasmid pZR2239 containing the genes controlled by their native promoter system was able to complement the mutant. Since the only difference between the complement strains was the promoter system used, the non-native promoter systems used in DR935(pZR2248) and DR935(pZR2243) are likely responsible for the failure of complementation. It is possible that the constitutive *glnA* promoter used in the unsuccessful complements did not function as optimally as the native promoters for either *aar* or *ado*.

These results indicate the autonomous regulation and perhaps differential expression of *aar* and *ado* are necessary for optimal functioning and/or interplay of the enzymes within the alkane biosynthetic pathway. Indeed, comparison of microarray experiments detailing expression of *aar* and *ado* in *Synechocystis* revealed that contrary fold changes occurred in the genes under many conditions (Klahn et al. 2014). Our results provide further evidence for the unique and differential expression of *aar* and *ado* as well as the importance of the genes’ independent expression for heptadecane production, consistent with the observation that the basal mRNA level of *alr5283* was approximately sixfold higher than that of *alr5284* in *Anabaena* 7120 (Flaherty et al. 2011).

Though translational regulation and post-translational regulation may also play important roles in heptadecane production in *Anabaena* 7120, our results suggest that the native promoter system has a central role in directing hydrocarbon production, and may in fact be vital. As previously suggested, the dual promoter system for *ado* may indicate different functions for *ado*, one of those being alkane production (Klahn et al. 2014). One commonality between both of the unsuccessful complement strains used in this study is that both placed *ado* under control of a non-native promoter (*glnA*). These results combined with the understanding of transcriptional regulation obtained in Klähn’s study further point to a potential divergence of ADO’s function in more than one cellular process. In addition, the results suggest a more complex understanding of the native promoter system and its role in directing ADO’s expression and incorporation into cellular metabolism. Future research may be aimed at elucidating the roles of the proximal and distal *ado* promoters and underscore any differences that exist between *ado* expressions when driven from either promoter.

### Potential role of heptadecane in cyanobacteria and its application in biofuel production

In this research, heptadecane was consistently found in the headspace of the cultures. Another study reported more than 80% of hydrocarbons produced by *Nostoc punctiforme* PCC 73102 were found outside the cells (Schirmer et al. 2010). In this study, under normal growth conditions (30 °C, normal light) growth of the mutant culture did not appear to be impacted by the loss of heptadecane production. Thus, under these conditions, though heptadecane is produced in small amounts, it is not required for survival and normal growth. However, it is possible that alkanes are required for response to certain stress conditions. Research has indicated that alkane production may be related to stress tolerance under various conditions, such as cold, high salinity, and high light (Berla et al. 2015; Bhadauriya et al. 2008; Kageyama et al. 2015; Takatani et al. 2015). In addition to abiotic stress responses, alkanes have also shown antibacterial activity in the cyanobacterium *Spirulina platensis* (Ozdemir et al. 2004). It is also possible that heptadecane has a function that is duplicated by another compound in the cell, such that when heptadecane is not produced, its vital function is still carried out.

With its high carbon content, heptadecane confers valuable fuel traits such as higher cetane number and oxidative stability, which are associated with long carbon chains and saturation (Quintana et al. 2011). Cyanobacteria present themselves as ideal fuel producers given their photosynthetic ability to convert CO_2_ to fuel using only the solar energy. Extraction processes often constitute 70–80% of production costs (Liu et al. 2011). Thus, in organisms which do not naturally secrete target compounds, further genetic engineering steps are required to enable product secretion from the cell (Liu et al. 2011). Our findings show that the extraction process is bypassed in hydrocarbon production by *Anabaena* 7120 since heptadecane was naturally secreted from the cells and captured from the headspace.

To enable large-scale commercialization of cyanobacteria fuel/chemical production systems, productivity and yield must be increased. As currently understood, alkanes are derived from fatty acids in cyanobacteria via a reduction-decarbonylation pathway. Fatty acids are produced to store energy in the cell. The balance in cells between storage and metabolism/growth is tightly controlled (Greenwell et al. 2010). Since fatty acids are the precursor to the alkanes we seek as fuel, the regulation of this balance must be explored. How do we circumvent this process to attain both growth and the fatty acid production associated with the storage state? Another study attempted to bypass the regulation by genetically engineering microalgae to increase lipid synthesis through over-expression of acetyl-CoA carboxylase. However, the change did not result in greater lipid production (Dunahay et al. 1996). Future work should focus on increasing our understanding of the regulatory mechanisms in lipid storage and cell growth.

## Additional file

**Additional file 1: Fig. S1.** Schematic illustration of pZR935 construction for knocking out *alr*5283 and *alr*5284 in *Anabaena* sp. strain PCC 7120.
