# Supplementary material for: Identification of two genes required for heptadecane production in a N2-fixing cyanobacterium Anabaena sp. strain PCC 7120
Source: AMB Express. 2018 Oct 13;8:167. doi: 10.1186/s13568-018-0700-6 (PMC6186262; doi:10.1186/s13568-018-0700-6)
Supplement: Supplementary file 1 — Additional file 1: Fig. S1. Schematic illustration of pZR935 construction for knocking out alr5283 and alr5284 in Anabaena sp. strain PCC 7120. [file 13568_2018_700_MOESM1_ESM.pdf]

AMB Express

Identification of Two Genes Required for Heptadecane Production in a N_2-fixing Cyanobacterium *Anabaena* sp. Strain PCC 7120

Jaimie Gibbons, Liping Gu*, Huilan Zhu, William Gibbons and Ruanbao Zhou*

Department of Biology and Microbiology, South Dakota State University, Brookings, SD 57007, USA

*Corresponding authors: ruanbao.zhou@sdstate.edu;

Tel:1-605-6885259 ; Fax : 605 688 5624

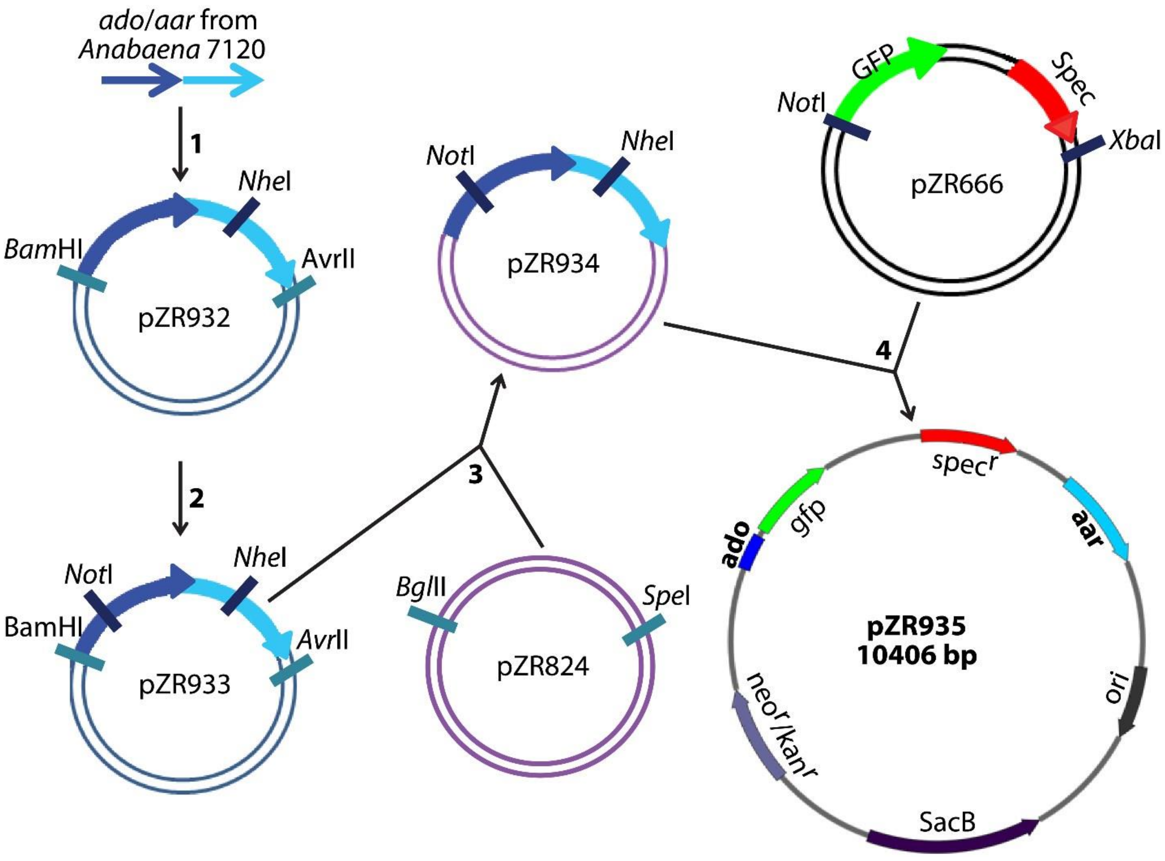

Fig. S1. Schematic illustration of pZR935 construction for knocking out *alr*5283 and *alr*5284 in *Anabaena* sp. strain PCC 7120.

Step 1: Amplified 2.7 kb fragment from *Anabaena* 7120 containing *alr*5283-84 using primers ZR241, 242. Cloned PCR product ligated to pCR2.1-TOPO vector to produce pZR932.

Step 2: Site directed mutagenesis using primers ZR243, 244 to introduce *Not*I site within *alr*5283 in pZR932, creating pZR933.

Step 3: Digestion of pZR933 with *Bam*HI and *Avr*II to obtain 2.7 kb *alr*5283-84 mutated sequence. Ligated this sequence to *Bgl*II and *Spe*I cut pZR824 vector, creating pZR934.

Step 4: *Not*I and *Xba*I cut out promoter-less GFP-Spec cassette from pZR666 ligated into *Not*I and *Nhe*I digested pZR934 to produce pZR935.
